# Unraveling the role of NLRP3 inflammasome in allergic inflammation: implications for novel therapies

**DOI:** 10.3389/fimmu.2024.1435892

**Published:** 2024-07-26

**Authors:** Hui-Fei Lu, Yi-Chi Zhou, Tian-Yong Hu, Dun-Hui Yang, Xi-Jia Wang, Dan-Dan Luo, Shu-Qi Qiu, Bao-Hui Cheng, Xian-Hai Zeng

**Affiliations:** ^1^ Zhuhai Campus of Zunyi Medical University, Zhuhai, China; ^2^ Department of Otolaryngology, Longgang Otolaryngology Hospital & Shenzhen Otolaryngology Research, Shenzhen, China; ^3^ Department of Gastroenterology, Beijing University of Chinese Medicine Shenzhen Hospital (Longgang), Shenzhen, China

**Keywords:** NLRP3 inflammasome, allergic diseases, IL-1β/IL-18 signaling, pyroptosis, therapeutic potential

## Abstract

Allergic diseases like asthma, allergic rhinitis and dermatitis pose a significant global health burden, driving the search for novel therapies. The NLRP3 inflammasome, a key component of the innate immune system, is implicated in various inflammatory diseases. Upon exposure to allergens, NLRP3 undergoes a two-step activation process (priming and assembly) to form active inflammasomes. These inflammasomes trigger caspase-1 activation, leading to the cleavage of pro-inflammatory cytokines (IL-1β and IL-18) and GSDMD. This process induces pyroptosis and amplifies inflammation. Recent studies in humans and mice strongly suggest a link between the NLRP3 inflammasome, IL-1β, and IL-18, and the development of allergic diseases. However, further research is needed to fully understand NLRP3’s specific mechanisms in allergies. This review aims to summarize the latest advances in NLRP3 activation and regulation. We will discuss small molecule drugs and natural products targeting NLRP3 as potential therapeutic strategies for allergic diseases.

## 1 Introduction

Allergic diseases, including asthma, allergic rhinitis (AR), and allergic dermatitis (AD), are type 2 immune response disorders caused by a complex interplay of genetic and environmental factors. These conditions are listed by the World Health Organization (WHO) as one of the three major chronic diseases requiring prevention and control in the 21st century (1). Their increasing prevalence burdens healthcare systems and patients, impacting quality of life (2, 3). Factors like air pollution, climate change, and lifestyle shifts are believed to contribute (4). Current treatment options, including allergen avoidance, medications, and immunotherapy, can be limited by the complex nature of allergic diseases. However, a deeper understanding of their pathogenesis has revealed potential therapeutic targets. Notably, the discovery of inflammasomes opens new avenues for targeted therapy strategies (5).

Inflammasomes, multiprotein complexes, are crucial regulators of inflammatory responses triggered by infection or cellular damage. They play a vital role in innate immunity (6). Pattern recognition receptors (PRRs) recognize pathogen-associated molecular patterns (PAMPs) and damage-associated molecular patterns (DAMPs) that trigger defense mechanisms to eliminate infection and/or repair damaged tissue (7, 8). Currently, PRRs mainly include Toll-like receptors (TLRs) and nucleotide-binding oligomeric domain-like receptors (NLRs). TLRs are located on the cell chamber and cell membrane, and NLRs are located in the cytoplasm. Inflammasomes are defined by their sensor proteins (PRRs), whose oligomerization forms a pre-caspase-1 activation platform in response to DAMPs or PAMPs (9). Activated Caspase-1 cleaves inactive forms of interleukin-1β (IL-1β) and IL-18 into their mature, pro-inflammatory counterparts, exacerbating disease (10–12). Inflammasome activation can also induce pyroptosis, a form of programmed cell death (13). While numerous inflammasomes exist (NLRP1, NLRP2, etc.) (14–17), NLRP3 has received significant attention due to its potential involvement in various diseases, including allergic diseases, chronic obstructive pulmonary disease (COPD), and cryopyrin-associated periodic fever syndromes (CAPS) (6, 13).

Mounting evidence from experimental studies suggests NLRP3 plays a critical role in the immune response to allergens, making it a promising therapeutic target for allergic diseases. NLRP3 inflammasome research is a dynamic field in immunology, with its function and regulation attracting significant interest. This review delves into recent NLRP3 research, exploring its activation and regulation mechanisms, its role in allergic diseases, and its potential as a novel therapeutic target.

## 2 The NLRP3 inflammasome: an overview

### 2.1 NLRP3 inflammasome composition

**Figure 1:**
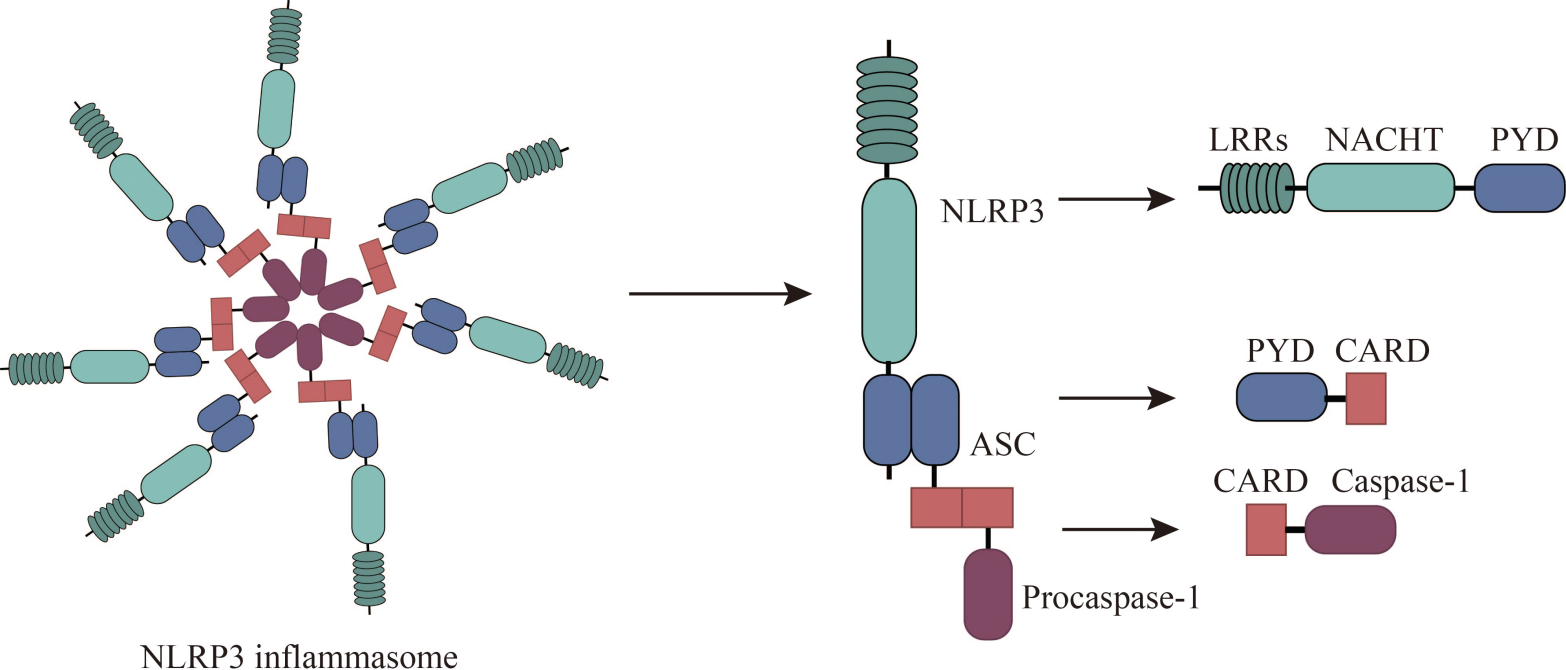
Composition of NLRP3 Inflammasomes. The NLRP3 inflammasome consists of NLRP3, associated speck-like protein (ASC), and procaspase-1. NLRP3 comprises an N-terminal pyrin domain (PYD), a central NACHT domain (nucleotide-binding oligomerization domain), and a C-terminal leucine-rich repeat domain (LRR). ASC comprises PYD and CARD (cysteine-aspartate-specific protease (caspase) recruitment domain) and interacts with NLRP3 and procaspase-1 through PYD-PYD and CARD-CARD, respectively.

NLRP3, a pivotal member of the NLR (nucleotide-binding oligomerization domain-like receptor) family, is increasingly recognized as a promising therapeutic target for a spectrum of conditions, including allergies (18). NLRP3 is expressed by a variety of cells, including neutrophils, macrophages, microglia, lymphocytes, epithelial cells, osteoblasts, neurons, and dendritic cells (19, 20). Structurally, NLRP3 is a multi-domain protein, encompassing a pyrin domain (PYD), a leucine-rich repeat (LRR) domain, and a nucleotide-binding domain (NACHT) (21). The NACHT domain comprises several subunits: a fish-specific NACHT-associated (FISNA) domain, a nucleotide-binding domain (NBD), two helical domains (HD1 and HD2), and a winged helix domain (WHD) (19). The LRR domain comprises multiple repeating units crucial for protein-protein interactions (19). The PYD, belonging to the death domain superfamily, exhibits a characteristic six-helix bundle structure (22). Activation of NLRP3 necessitates ATP binding to its NACHT domain, which possesses ATPase activity; mutations in this domain can result in loss of function (23). The PYD also contributes to downstream signaling through homotypic interactions, while LRR domains primarily mediate protein-protein interactions (24, 25). Upon exposure to allergens, NLRP3 initiates a cascade of events by interacting with the adaptor protein ASC to form an inflammasome complex, which subsequently activates caspase-1, a key enzyme in the inflammatory response (26–28). The NLRP3 inflammasome comprises the sensor (NLRP3), the adaptor (ASC), and the effector (caspase-1) (18). This complex orchestrates the maturation and release of cytokines such as IL-1β and IL-18, as well as the activation of gasdermin D (GSDMD), which mediates cytokine release and pyroptosis (19, 29). Caspase-1 itself comprises an N-terminal CARD domain and two catalytic subunits, p10 and p20 (30). (**Figure 1**) Responsive to various infectious agents including bacteria, viruses, fungi, and parasites, the NLRP3 inflammasome, when activated, facilitates the release of inflammatory mediators like IL-1β, IL-18, high mobility group box 1 protein (HMGB1), leukotrienes, and prostaglandins (31–34).

### 2.2 Mechanism of NLRP3 inflammasome activation

Recent studies have widely acknowledged that the activation of NLRP3 inflammasomes involves two distinct stages: priming and activation (35). In the quiescent cellular state, basal levels of NLRP3 and IL-1β are deemed insufficient to initiate inflammasome activation. Consequently, the priming process becomes imperative for NLRP3 inflammasome activation. During the priming phase, upregulation of inflammasome components NLRP3, caspase 1, and pro-IL-1β occurs through signaling pathways mediated by TLRs, NOD2, and cytokine receptors (IL1R, or TNFR) in response to PAMPs and DAMPs such as LPS or cytokines like TNF and IL-1β (18). Additionally, priming regulates post-translational Modifications (PTMs) of NLRP3, including ubiquitination and phosphorylation, crucial for the activation and inhibition of NLRP3 inflammasome (36). Microbial ligands and endogenous cytokines such as TNF-α or IL-1β activate the NF-κB pathway by stimulating TLR, TNF, or IL-1 receptors during priming, leading to the transcription of NLRP3 and pro-IL-1β genes (37, 38).

Subsequent to the priming stage, activated NLRP3 forms inflammasomes alongside ASCs and pro-caspase-1, culminating in the complete activation of NLRP3 inflammasomes. The activation of NLRP3 inflammasomes encompasses a diverse array of agonistic signals, resulting in a complex activation mechanism. Recent studies propose that NLRP3 inflammasomes can be activated through three different signaling pathways, including canonical NLRP3 inflammasome activation, non-canonical NLRP3 inflammasome activation, and alternative NLRP3 inflammasome activation (35, 39).

#### 2.2.1 Canonical NLRP3 inflammasome activation

##### 2.2.1.1 Ion flux

Canonical NLRP3 inflammasome activation has implicated ion flux (e.g., K^+^ efflux, Cl^-^ efflux, Na^+^ influx, and Ca^2+^ mobilization), mitochondrial dysfunction, reactive oxygen species (ROS) release, and lysosomal destabilization (40). These pivotal activation pathways are considered upstream signals for NLRP3 stimulation-induced inflammasome assembly and activation. Notably, these pathways ultimately converge on potassium efflux from the cell. Muñoz-Planillo et al. demonstrated that the only consistent activity induced by all NLRP3 agonists was the permeation of the cell membrane by K^+^ and Na^+^, with a reduction in intracellular K^+^ concentration being sufficient to activate NLRP3 (41). Presently, numerous agonists can induce potassium efflux, including extracellular ATP (via the non-selective cation channel receptor P2X7), particulate matter (e.g., aluminum hydroxide, silica, and calcium pyrophosphate crystals), and nigericin (an ionophore) (41–43). However, recent findings have revealed that NEK7 (NIMA-related kinase 7), a component of the NLRP3 inflammasome, directly binds to the NLRP3 protein, with K^+^ efflux being necessary for the assembly of the NLRP3 inflammasome (44, 45). He et al. illustrated that NEK7 is an indispensable protein mediating NLRP3 inflammasome assembly and activation downstream of potassium flux, with the interaction between NLRP3 and NEK7 being influenced by extracellular potassium concentrations, dissipating when extracellular potassium concentrations exceed 50 mM (45). Zeng et al. demonstrated *in vitro* experiments in mouse model with Dextran sodium sulfate (DSS)-induced colitis that DSS enhanced NLRP3 inflammasome activation in macrophages by enhancing KCa3.1-mediated K^+^ efflux (46). These studies suggest that a decrease in intracellular K^+^ concentrations is the single most necessary and sufficient event for the activation of caspase-1 and NLRP3 inflammasome activation. In addition, there is still evidence that Cl^-^efflux also plays an important role in inflammasome activation. Tang et al. showed that chloride intracellular channels (CLICs) play a downstream role in the potassium efflux-mitochondrial ROS axis to promote NLRP3 inflammasome activation. Subsequently, NLRP3 agonists induce potassium efflux, causing mitochondrial damage and ROS production, and mitochondrial ROS induces the transfer of CLICs to the plasma membrane to induce chloride efflux, promoting NEK7-NLRP3 interaction, inflammasome assembly, caspase-1 activation and IL-1β secret (47). Similarly, Green et al. delineated the roles of K^+^ efflux and Cl^-^ efflux by independently inhibiting either K^+^ or Cl^-^ efflux during NLRP3 activation, concluding that K^+^ efflux drives NLRP3 oligomerization, while Cl^-^ efflux facilitates ASC aggregation during NLRP3 inflammasome formation (48). Furthermore, Ca^2+^ mobilization is also a key upstream event for NLRP3 activation. It has been reported that elevated extracellular calcium levels stimulate G protein-coupled calcium-sensing receptors to catalyze the production of inositol-1,4,5-triphosphate through phospholipase C, induce the release of Ca^2+^ from endoplasmic reticulum storage, and then activate the NLRP3 inflammasome (49). Moreover, studies have found that excessive or sustained ca^2+^ uptake promotes mitochondrial dysfunction, which is characterized by increased mitochondrial ROS (mtROS) production and altered mitochondrial permeability, which ultimately leads to mitochondrial rupture and the release of mtROS and mitochondrial DNA (mtDNA) into the cytoplasm to activate the NLRP3 inflammasome (50). In addition, studies have shown that the regulation of Na^+^ influx plays a role in NLRP3 activation, but is not necessary for NLRP3 activation (41). Some studies suggest that Na^+^ influx may regulate NLRP3 activation by reducing the decrease in K^+^ in cells (51). Moreover, there are also other ion fluxes involved in the activation of NLRP3 inflammasomes, such as Mg^2+^, MN^2+^, Zn^2+^, Fe^2+^, etc (51). Currently, the precise mechanism of ion flux activation in the NLRP3 inflammasome remains incompletely understood, necessitating further exploration of the role of ion flux in NLRP3 activation mechanisms.

##### 2.2.1.2 Lysosomal destabilization

Phagocytosis of various exogenous and endogenous particulate substances, such as monosodium urate (MSU), calcium pyrophosphate dihydrate (CPPD), silica, asbestos, and the heavy subunit of ferritin (FTH), has been found to activate NLRP3 inflammasomes (52–55). The process begins when lysosomal swelling and damage, caused by the ingestion of these particles, result in lysosomal rupture. This rupture releases the particles into the cytoplasm, which in turn promotes the assembly and activation of NLRP3 inflammasome (52). Hornung et al. have directly demonstrated that lysosomal damage can lead to the activation of the NLRP3 inflammasome through the release of the dipeptide-leucine-leucine methyl ester (Leu-Leu-OMe) (52). Furthermore, lysosomal disruption can activate the NLRP3 inflammasome via the release of Ca^2+^ ions, which then trigger the inflammasome’s activation (50). Additionally, lysosomal disruption induced by Leu-Leu-OMe and NLRP3 particle stimulation can activate K^+^ efflux, although the exact mechanism linking lysosomal rupture to K^+^ efflux is not yet fully understood (41). Studies have also indicated that the release of cathepsin B from lysosomes can directly activate the NLRP3 inflammasome and induce mitochondrial dysfunction, further contributing to NLRP3 activation (56). Conversely, suppression of phagosomal acidification or cathepsin B activity has been shown to weaken NLRP3 activation (57). The enzyme inhibitor Ca074Me has been reported to inhibit NLRP3 inflammasome activation by various cathepsins (58). Therefore, the mechanisms by which lysosomal enzyme release triggers NLRP3 activation warrant further investigation.

##### 2.2.1.3 Mitochondrial dysfunction and reactive oxygen species

Mitochondrial dysfunction has also emerged as a critical event in NLRP3 inflammasome activation. Damaged mitochondria release signals such as mtDNA and mtROS, which can activate NLRP3, leading to caspase-1 activation and the release of the cytokine IL-1β (59, 60). mtROS production is recognized as a key factor in NLRP3 inflammasome activation (61). Research has shown that mtROS can both directly regulate inflammasome assembly and indirectly influence inflammasome activity by affecting cytoplasmic proteins (62). Zhou et al. have found that mtROS can mediate the dissociation of thioredoxin-interacting protein (TXNIP) from thioredoxin, leading to NLRP3 activation and IL-1β release (63). Additionally, ROS-induced activation of the NF-κB pathway can promote the expression of pro-inflammatory factors, including pro-IL-1β, TNF-α, and IL-6, as well as indirectly enhance NLRP3 expression (64). Hou et al. have demonstrated that CD36, a class B scavenger receptor, can activate the NLRP3 inflammasome via the mtROS pathway in a mouse model of diabetic nephropathy (65). Furthermore, mtDNA can also initiate NLRP3 inflammasome activation. Shimada et al. have proposed that, under NF-κB activation, ATP can induce mitochondrial dysfunction and apoptosis, leading to the release of oxidized mitochondrial DNA (OX-mtDNA), which then activates NLRP3 inflammasomes (60). Ding et al. have suggested that damaged mtDNA can trigger autophagy and NLRP3 inflammasome activation, with LOX-1 potentially playing a pivotal role in this process (66). Studies using human THP-1 macrophages and primary macrophages have shown that LOX-1-mediated autophagy and mtDNA damage are important in NLRP3 inflammasome activation during inflammatory diseases (66). Qiu et al. have noted that intracellular and extracellular mtDNA have distinct roles in NLRP3 inflammasome activation: intracellular mtDNA, being more prone to oxidation, can directly bind to NLRP3 and activate the inflammasome, while extracellular mtDNA acts as a DAMP in the initiation and activation of the NLRP3 inflammasome (67). Shimada et al. have also found that mtDNA is rapidly released into the cytoplasm upon stimulation by various NLRP3 activators, with oxidized mtDNA specifically activating NLRP3 inflammasomes, and non-oxidized mtDNA preferentially stimulating AIM2 inflammasomes (60). Collectively, these findings reveal the pivotal role of both mtROS and mtDNA in NLRP3 inflammasome activation. Moreover, mitochondria are increasingly recognized as docking sites for inflammasome assembly, with at least three proteins—cardiolipin, a mitochondrial protein, and mitofusin2—serving as potential junctions between NLRP3 and mitochondria (18). The precise role of mitochondrial dysfunction in NLRP3 activation requires further exploratio.

**Figure 2:**
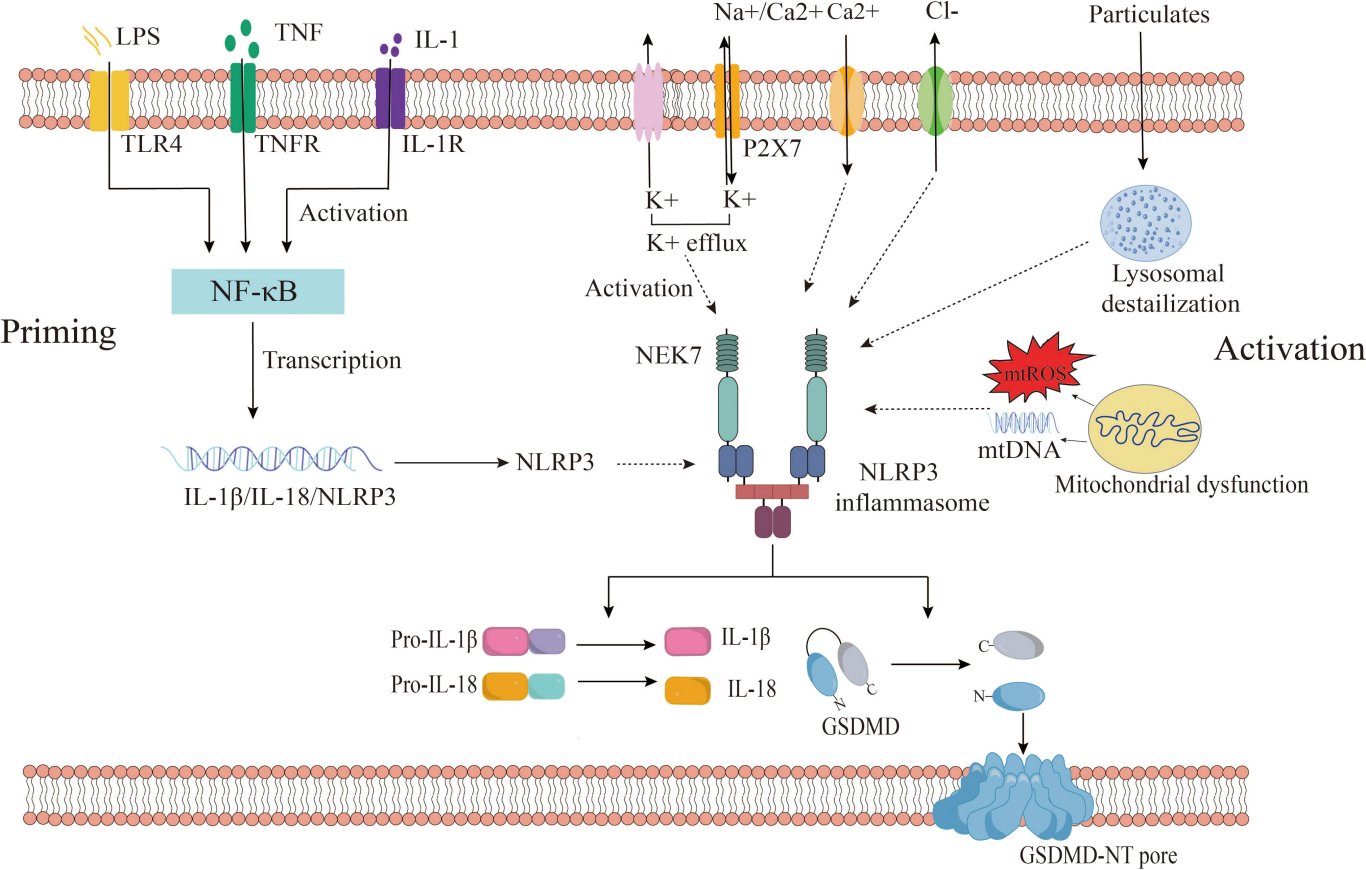
Canonical NLRP3 inflammasome activation. the activation of NLRP3 inflammasomes consists of two stages: priming and activation. Among them, the priming phase involves PAMPs/DAMPs or cytokine-induced NF-kB activation, upregulating gene expression of NLRP3, IL-1β, IL-18. The activation phase is provided by various PAMPs and DAMPs. They activate multiple upstream signaling events, such as K^+^ efflux, Ca^2+^ mobilization, Na^+^ influx, Cl^-^ efflux, mtROS production, release of oxidized mtDNA, and lysosomal damage, promote NLRP3 inflammasome assembly and activation, and enable caspase-1 activation. Activated caspase-1 then cleaves pro-IL-1β and pro-IL-18 to obtain mature pro-inflammatory factors IL-1β and IL-18, which are involved in inflammatory responses. Active caspase-1 also cleaves GSDMD, which is divided into C-terminus and N-terminus of GSDMD, and free GSDMD-N oligomerizes on the membrane to form pores, inducing pyroptosis. GSDMD, gasdermin D; TLR, Toll-like receptor; IL-1β, interleukin-1β; IL-18, interleukin-18; P2X7, P2X purinoceptor 7; ROS, reactive oxygen species; mtDNA, mitochondrial DNA.

In summary, NLRP3 activation is triggered by a variety of cellular and molecular events, including ion fluxes, lysosomal destabilization, and mitochondrial dysfunction. Recent studies have also shown that different NLRP3 stimuli can lead to the breakdown of the anti-Golgi network (TGN), which in turn activates NLRP3 (68). NLRP3 is recruited to dispersed TGN (dTGN) through an ionic interaction between its conserved polyhedral region and negatively charged phosphatidylinositol 4-phosphate (PtdIns4P) on dTGN (68). The dTGN then serves as a scaffold for NLRP3 aggregation into multiple puncture sites, leading to the polymerization of the adaptor protein ASC and the activation of downstream signaling cascades. The interaction between NLRP3 and PtdIns4P on dTGN is disrupted when NLRP3 aggregation and downstream signaling are blocked. These findings indicate that the recruitment of NLRP3 to dTGN is an early and common cellular event that results in NLRP3 aggregation and activation in response to diverse stimuli (68). Given the multitude of NLRP3 activation mechanisms, further research is necessary to elucidate the precise mechanisms, including the role of K^+^ efflux and other pathways, in NLRP3 inflammasome activation (**Figure 2**).

#### 2.2.2 Non-canonical NLRP3 inflammasome activation

Beyond the canonical caspase-1 pathway, recent research reveals an alternative way to activate the NLRP3 inflammasome, involving caspase-4/5 in humans or caspase-11 in mice (69, 70). This noncanonical inflammasome activation is initiated by the intracellular detection of LPS by murine caspase-11 or human caspase-4/5, which sets off a proteolytic cascade. This cascade culminates in the cleavage of GSDMD and the subsequent activation of the NLRP3 inflammasome (71). The outer membrane LPS of Gram-negative bacteria may serve as a pivotal activator of noncanonical inflammasomes. Further research has demonstrated that human caspase-4/5 and murine caspase-11 can directly and specifically bind to LPS and lipid A, leading to caspase oligomerization and autoproteolysis (72). Studies have confirmed that caspase-11 and its human counterparts, caspase-4 and caspase-5, recognize intracellular LPS from gram-negative bacteria during macrophage-driven inflammatory responses. This direct recognition rapidly triggers oligomerization of caspase-11/4/5, initiating pyroptosis and the secretion of inflammatory cytokines, including IL-1β and IL-18 (73). Upon activation, caspase-4/5/11 cleaves GSDMD at Asp276 (in mice) and Asp275 (in humans), generating an N-terminal cleavage product (GSDMD-NT) that instigates pyroptosis and the release of pro-inflammatory cytokines such as interleukin-1β (74). GSDMD-NT interacts with cardiolipin, phosphatidylinositol phosphate, and phosphatidylserine on the plasma membrane, causing the formation of GSDMD pores approximately 21 nm in diameter. This leads to osmotic imbalance and cell pyroptosis (75). Interestingly, this process also promotes the activation of the K^+^ efflux-dependent canonical NLRP3 inflammasome pathway, further activating caspase-1 and facilitating the secretion of IL-1β (74, 76). This suggests a crosstalk between canonical and non-canonical NLRP3 inflammasome activation pathways. Additionally, studies have found that oxidized phospholipids derived from arachidonic acid can enhance caspase-11-mediated pro-inflammatory responses and stimulate IL-1β release, albeit without the hyperactivation observed with caspase-1 (77).

#### 2.2.3 Alternative NLRP3 inflammasome activation

**Figure 3:**
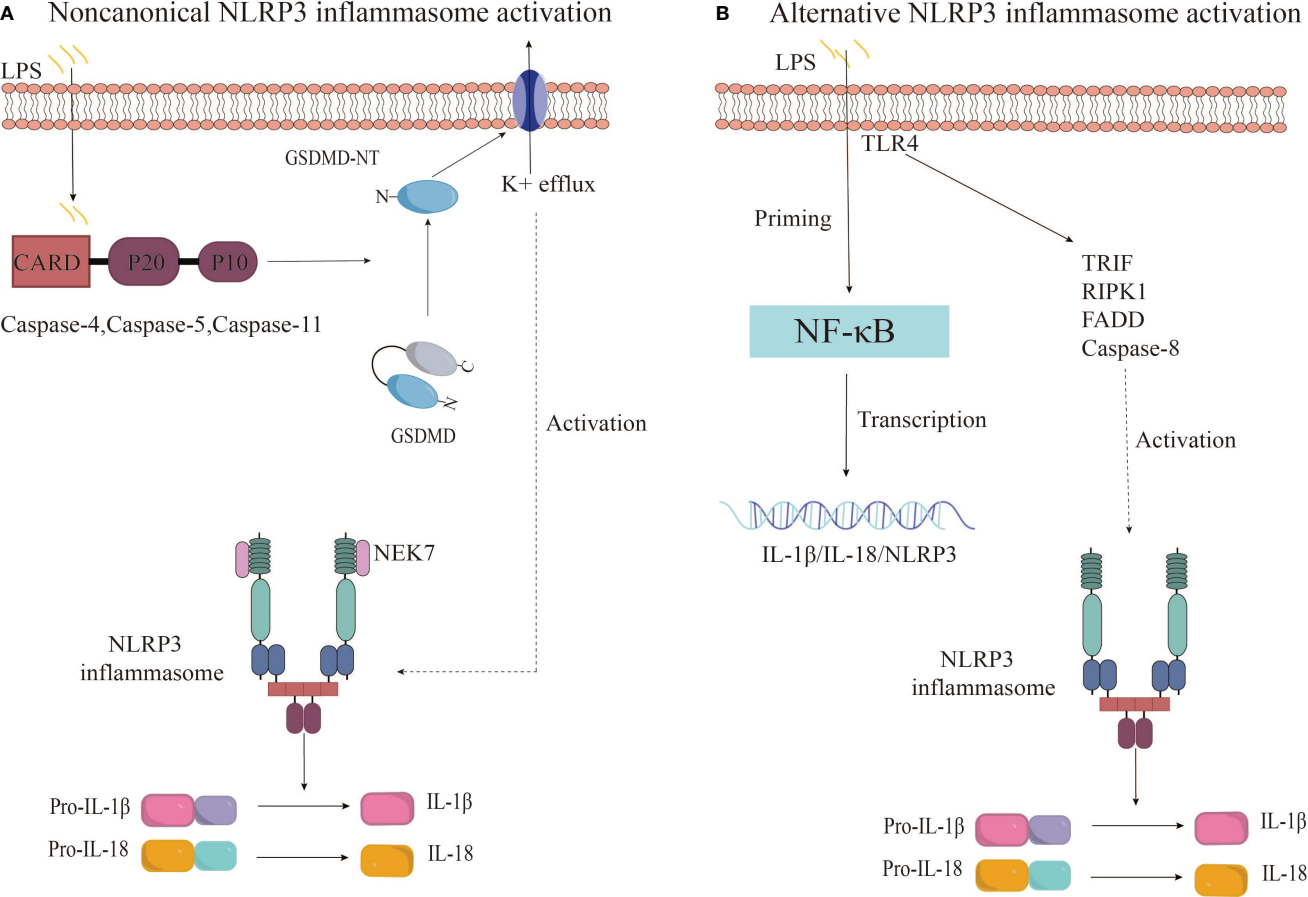
Noncanonical and alternative NLRP3 inflammasome activation pathways. **(A)** The noncanonical inflammasome activation is initiated by the activation of caspase-4/5/11 upon recognition of intracellular LPS, leading to GSDMD cleavage and pyroptosis. This results in potassium efflux and subsequently triggers the canonical NLRP3 inflammasome pathway. **(B)** The alternative NLRP3 inflammasome activation in monocytes is characterized by LPS-induced activation of the TRIF-RIPK1-FADD-CASP8 signaling pathway upstream of NLRP3 through TLR4 receptors, culminating in the secretion of IL-1β and IL-18.

Research has uncovered an alternative activation pathway for the NLRP3 inflammasome, which is mediated by the TLR4 signaling pathway and does not require secondary signaling (78). Gaidt et al. proposed that this alternative inflammasome activation is propagated through a TLR4-TRIF-RIPK1-FADD-CASP8 signaling cascade upstream of NLRP3 (79). Notably, the involvement of this signaling cascade is specific to alternative inflammasome activation and does not apply to the classical NLRP3 activation pathway (79). In human monocytes, this pathway relies on NLRP3-ASC-caspase-1 signaling but lacks the hallmark features of classical inflammasome activation, such as pyroptosis formation and K^+^ efflux dependence (78, 79). Unterberger et al. demonstrated that TLR-induced IL-1β release is mediated by constitutively active caspase-1 and caspase-8, and that TLRs can activate caspase-1 and promote IL-1β secretion independently of RIPK1 kinase activity (78). However, the precise mechanism by which caspase-8 activates NLRP3 during alternative inflammasome activation is not yet clear. Further research is necessary to delineate the relationship between caspase-8 and NLRP3 within the alternative activation pathway (**Figure 3**).

## 3 Post-translational modifications

PTMs involve the covalent processing of specific proteins after their translation, which can be either reversible or irreversible. These modifications occur at the amino acid side chains, the C-terminus, or the N-terminus and involve the addition of particular chemical groups, proteins, carbohydrates, or lipids to the amino acid side chains, or through enzymatic bonding. This process alters protein structure, enhancing the functional diversity of proteins (80). PTMs, often enzyme-catalyzed, are integral to the pathophysiological processes of various diseases (81). A multitude of studies suggest that the intracellular levels of the NLRP3 protein are pivotal in the canonical activation of the NLRP3 inflammasome. Consequently, PTMs play a significant role in the activation and functional expression of NLRP3 inflammasomes. Several PTMs of NLRP3 have been reported, including phosphorylation, acetylation, ubiquitination, palmitoylation, and sumoylation (82–84), with ubiquitination and phosphorylation being the most extensively studied (80). Ubiquitination of NLRP3, involving K48-, K63-, and K27-linked chains, is known to regulate NLRP3 inflammasome activation (80). While K48-linked ubiquitination inhibits activation through NLRP3 degradation, K63-linked ubiquitination typically facilitates it (80). The role of K27-linked ubiquitination in NLRP3 inflammasome regulation varies. Wang et al. demonstrated that the transcriptional coactivator YAP interacts with NLRP3, maintaining its stability by preventing the association with the E3 ligase β-TrCP1, which targets NLRP3 for degradation at lys380 through K27-linked ubiquitination (85). Conversely, the E3 ubiquitin ligase HUWE1 mediates K27-linked polyubiquitination of AIM2, NLRP3, and NLRC4, promoting inflammasome assembly and sustained caspase-1 activation (86). Phosphorylation and dephosphorylation of NLRP3 are also crucial for NLRP3 inflammasome regulation. Phosphorylation modulates protein function, turnover, and subcellular localization by altering conformation or protein interactions (80). Song et al. confirmed that JNK1-mediated phosphorylation at NLRP3 S194 is essential for NLRP3 deubiquitination and subsequent inflammasome assembly. In a mouse model with a knockout of the NLRP3-S194A gene, inflammasome activation was disrupted, indicating that inhibiting NLRP3 phosphorylation can limit its activation (87). Phosphorylation of NLRP3, particularly at S194, has been observed during the priming phase in bone marrow-derived macrophages (BMDMs) and in primary human monocytes under various stimuli, such as LPS, suggesting it as an early event in the priming process (87). Mortimer et al. showed that protein kinase A (PKA) phosphorylates Ser295 in NLRP3, inhibiting the ATPase function of the NACHT domain and negatively regulating NLRP3 (88). Additionally, phosphorylation at human Ser5 (equivalent to mouse Ser3) inhibits NLRP3 inflammasome activation as it resides at the PYD-PYD interface, with dephosphorylation by protein phosphatase 2A (PP2A) being necessary for inflammasome activation following an additional signal (89). But Ser5 must be dephosphorylated by PP2A to allow the inflammasome to activate after an additional activation signal (89). Beyond ubiquitination and phosphorylation, other PTMs significantly influence NLRP3 regulation. Palmitoylation, a reversible lipid modification primarily catalyzed by the zinc-containing DHHC-type palmitoyltransferases, has been shown to promote NLRP3 degradation through chaperone-mediated autophagy, thereby inhibiting NLRP3 inflammasome activity (90–93). Specifically, zDHHC12-mediated palmitoylation targets NLRP3 for degradation, with C844 identified as the principal palmitoylation site (93). Furthermore, acetylation of NLRP3 has been implicated in inflammasome activation, with lys24 acetylation promoting NLRP3 oligomerization and activation (82). The lysine acetyltransferase 5 (KAT5) enhances NLRP3 inflammasome activation by regulating NLRP3 acetylation and oligomerization, with the KAT5 inhibitor NU 9056 significantly inhibiting NLRP3 inflammasome activation (82). Sumoylation also contributes to the regulation of NLRP3 inflammasomes, with sumoylation suppressing NLRP3 activation. NLRP3 sumoylation by the SUMO E3-ligase MAPL is counteracted by stimulation-dependent desumoylation by SENP6 and SENP7, promoting NLRP3 activation. Disruption of NLRP3 sumoylation, either by mutation of SUMO acceptor lysines or depletion of MAPL, results in enhanced caspase-1 activation and IL-1β release (94). Additionally, the E3 SUMO ligase TRIM28 enhances NLRP3 inflammasome activation by promoting NLRP3 expression and sumoylation, thereby inhibiting NLRP3 ubiquitination and proteasomal degradation (83).

In summary, NLRP3 must undergo multiple PTMs to achieve full activity. Further research is needed to elucidate the mechanisms of PTMs in regulating NLRP3 inflammasome activity. Understanding and targeting the PTM process could reveal novel therapeutic approaches for disease treatment.

## 4 The role of NLRP3 in allergic diseases

### 4.1 Mechanism of allergic diseases involving NLRP3

Allergic diseases, characterized by IgE-mediated type 2 inflammation, are complex and influenced by genetics, environment, microbiome, and immune function (1). Their high recurrence rate and limited treatment options pose significant burdens on patients (1). Research has shed light on the role of immune cells like Th2 cells, innate lymphoid cells (ILCs), dendritic cells, mast cells, and others in allergic disease development (95, 96). When allergens disrupt the mucosal barrier, epithelial cells release alarmins (IL-33, IL-25, TSLP) that, via pathways like NF-κB, AP-1, and STAT5, activate Th2 cells and ILCs. These cells produce type 2 cytokines (IL-4, IL-5, IL-9, IL-13) leading to acute allergic reactions (96). In susceptible individuals, allergens presented by dendritic cells and ILC2 activation drive the development of Th2 cells from naive T cells. IL-4 induces B cells to differentiate into plasma cells, producing IgE antibodies that bind to high-affinity receptors (FcϵRIs) on mast cells and basophils, sensitizing the body (97). Upon re-encountering the allergen, IgE triggers mast cell and basophil activation and degranulation, leading to a type 2 immune effector response.

**Figure 4:**
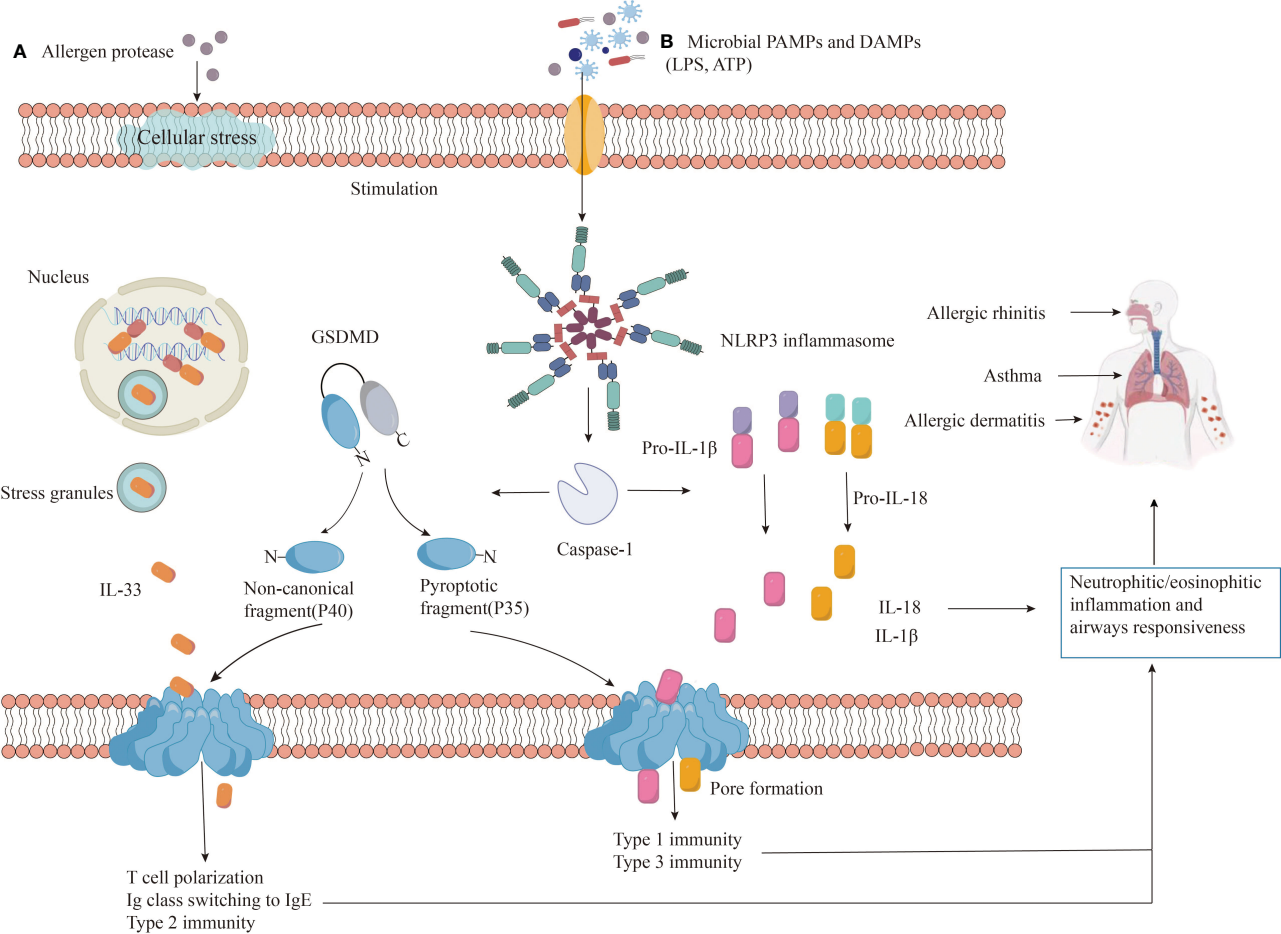
The role of NLRP3 in allergic diseases. Exposure to **(A)** allergens or **(B)** danger signals (PAMPs/DAMPs) triggers NLRP3 protein to undergo auto-oligomerization, forming an inflammasome complex. The assembled inflammasome activates caspase-1, which cleaves pro-inflammatory precursor molecules (pro-IL-1β and pro-IL-18) into their mature, active forms (IL-1β and IL-18). Additionally, caspase-1 also cleaves GSDMD protein, releasing the N-terminal fragment (GSDMD-N) that forms pores in the cell membrane, facilitating IL-33 release and initiating a type 2 immune response. The release of active IL-1β and IL-18, coupled with GSDMD-N-induced pyroptosis, contribute to inflammation, infiltration of neutrophils and eosinophils, airway hyperresponsiveness, and worsened disease severity.

However, inflammasome formation and pyroptosis can worsen allergic reactions. Microbial infections, pollutants, and allergens can be recognized by PRRs, leading to changes in ion concentration, mitochondrial dysfunction, and reactive oxygen species production. These factors, along with lysosomal damage, can initiate NLRP3 inflammasome assembly and caspase-1 activation. Activated caspase-1 cleaves pro-IL-1β and pro-IL-18 into mature IL-1β and IL-18, promoting inflammation. Additionally, it cleaves GSDMD, allowing its N-terminal fragment (GSDMD-N) to form pores in the cell membrane, leading to the release of cellular contents (including IL-33, IL-1β, and IL-18) and pyroptosis, further amplifying the inflammatory response (**Figure 4**).

### 4.2 The role of NLRP3 in allergic diseases

#### 4.2.1 NLRP3, IL-1β and IL-18 in asthma

Asthma is a chronic inflammation of the airways involving a variety of cells, especially mast cells, eosinophils and T lymphocytes, with the main symptoms being dyspnea, wheezing, cough and chest tightness, etc., which is an interaction between the innate immune system and the adaptive immune system (98). Numerous studies implicate NLRP3 inflammasomes, IL-1β and IL-18 in the development of asthma. Sawada et al. found that overexpression of IL-18 protein in the lungs induced type 1 and type 2 cytokine and airway inflammation in a mouse model of ovalbumin-induced and led to increased airway hyperresponsiveness through CD4^+^ T cells and IL-13 in asthma (99). In addition, Wang et al. observed an increased proportion of Th2, Th17, IL-18^+^, IL-18^+^ Th2 and IL-18^+^ Th17 cells in blood CD4^+^ T cells of asthma patients, and found that Th2 cells responded to allergen stimulation by expressing excessive IL-18 and IL-18Rα in an ova-induced mouse model of asthma (100). Similarly, many studies have shown high levels of IL-1β in bronchoalveolar lavage fluid (BALF), sputum, and serum. Additionally, Mahmutovic et al., in a mouse model of asthma exacerbations, validated that the expression of neutrophil cytokines and the release of major Th2 upstream cytokines depended on IL-1β signaling for the manifestation of exacerbated inflammation (101). However, studies have shown that increased NLRP3-inflammasome-mediated IL-1β responses correlate with neutrophilic asthma severity (102). Another study using a murine model of asthma reported that genetic `disruption of the IL-1 signaling pathway could significantly attenuate allergic inflammatory responses (103). Furthermore, the involvement of the NLRP3 inflammasome in the development and exacerbation of allergic asthma has been demonstrated. Ma et al. demonstrated that house dust mites (HDMs) extracts inhalation in mouse models activated NLRP3 inflammasomes in the lungs, specifically inducing the maturation of caspase-1 and IL-1β in alveolar macrophages (AMs), a critical factor in pulmonary type II immune response inflammation and tissue damage in asthma (104). Kim et al. found that increased NLRP3, caspase-1, and IL-1β responses aggravate steroid-resistant neutrophil inflammation and airway hyperresponsiveness in a mouse model of chlamydia and Haemophilus respiratory tract infection mediated and ovalbumin-induced severe steroid-resistant allergic asthma (105).

#### 4.2.2 NLRP3, IL-1β and IL-18 in AR

AR is a multifactorial disease caused by a combination of genetic and environmental factors, and the main symptoms are episodic sneezing, runny nose and nasal congestion. NLRP3 inflammasomes also play an important potential role in the occurrence and development of AR. Shi et al. isolated peripheral blood mononuclear cells (PBMCs) and monocytes/macrophages from persistent moderate-to-severe AR patients and healthy controls and stimulated with LPS. They observed that the mRNA expression levels of NLRP3 and IL-1β and the levels of IL-1β produced in monocytes/macrophages and PBMCs of AR patients were significantly upregulated compared to healthy controls (106). Hu et al. showed that in experiments with a mouse model of OVA-induced allergic rhinitis, it was found that macrophage depletion led to a decrease in IgE levels, and that IL-18 released by NLRP3-mediated pyroptosis induced Th1/Th2 differentiation in AR models (107). This comprehensive study reveals the critical role of NLRP3-mediated immunomodulation and macrophage pyroptosis in the regulation of Th1/Th2 homeostasis in OVA-induced AR. Furthermore, Yang et al. demonstrated that in a model of AR, NLRP3-deficient mice had attenuated inflammation and nasal mucosal damage compared with wild-type animals. This was associated with reduced ASC speck accumulation and pyroptosis. This implicates that the NLRP3 inflammasome and its downstream effects aggravate AR in this murine model (108). However, the specific downstream effect(s) responsible for the NLRP3-mediated enhancement of AR remain unclear. It is uncertain whether ASC speck formation, pyroptosis, or the reduction of IL-1β and IL-18 levels is the key driver, as these effects have not been experimentally isolated. Further investigation is required to elucidate the precise mechanism.

#### 4.2.3 NLRP3, IL-1β and IL-18 in AD

AD is an inflammatory skin disease caused by a combination of genetic and environmental factors, as well as epidermal barrier dysfunction and skin microbiota disorders. Previous studies have shown that HDM extracts stimulate keratinocytes to recruit NLRP3, ASC, and caspase-1 to the perinuclear region and induce inflammasome activation (109). This process results in the secretion of IL-1β and IL-18 in an inflammasome-dependent manner. These studies suggest a link between increased NLRP3 expression and AD. Kim et al.’s study using a mouse model of AD showed that Moringa concanensis l significantly inhibited IL-1β production by downregulating NLRP3 inflammasome activation, ameliorating AD symptoms, and implicating IL-1β in AD pathogenesis (110). In addition, NLRP3 can also play an independent role in AD, where NLRP3 interacts with the transcription factor IPF4 and binds to IL-33-specific promoters in the nucleus of keratinocytes, resulting in increased IL-33 secretion and worsening of AD (111).

In summary, the activation of NLRP3 can not only act on the development of allergic diseases, e.g., by inducing Th2 cell differentiation, but also aggravate the disease and promote the deterioration of cell and organ function. Furthermore, the activated NLRP3 inflammasome promotes the expression and regulation of various inflammatory factors, including IL-6, IL-17, and IL-33, participating in allergic disease development (106, 111, 112).

### 4.3 NLRP3 mediates pyroptosis in allergic diseases

Recent studies have highlighted the role of NLRP3-mediated pyroptosis in the development of allergic diseases. Pyroptosis is a type of programmed cell death (PCD) characterized by cell expansion until the cell membrane ruptures, resulting in the release of intracellular contents and triggering a strong inflammatory response. Activation of NLRP3 inflammasomes has been confirmed to be the central mediator of pyroptosis. Upon activation, caspase-1/4/5/11 cleaves the GSDMD protein, resulting in the n-terminal oligomerization of the GSDMD and the formation of pores in the cell membrane. The inflow of water through these pores causes the cell to swell and dissolve, eventually leading to the rupture of the plasma membrane. This process results in the release of inflammatory mediators such as IL-1β and IL-18 and aggravates inflammation (113). Yang et al. used wild-type and NLRP3 knockout mice to construct an OVA-induced AR models, and the experimental results showed that the production of IL-1β and the activation of inflammasomes were increased, and the activation of NLRP3 could promote AR progression and aggravate the inflammatory response and epithelial pyroptosis in AR mice (108). Similarly, Wang et al. confirmed that nonylphenol (NP) exposure can exacerbate inflammatory symptoms by activating NLRP3 inflammasome and GSDMD-mediated pyroptosis of nasal mucosal cells in an OVA-induced AR mouse model (114). Zhuang et al. studied by establishing a mouse model of toluene diisocyanate (TDI)-induced experimental asthma, in which experimental data showed that bronchial epithelial cell pyroptosis was induced by NLRP3 inflammasome activation and cleavage of GSDND, to exacerbate TDI-induced airway inflammation and hyperresponsiveness in asthma (115). The involvement of immune cell pyroptosis in the pathogenesis of allergic diseases is also been studied. For example, Hu et al. revealed that increased macrophage pyroptosis influences T cell differentiation in AR mouse models, with IL-18 released through NLRP3-mediated pyroptosis driving Th2 differentiation and exacerbating the allergic inflammatory response (107). Qiao et al. discovered aberrant activation of GSDMD-N-mediated pyroptosis in CD11c^+^ DCs from the spleen, nasal mucosa, and nasal drainage lymph nodes of AR mice, with pyroptosis of BMDCs showing dose-dependent morphological abnormalities, including numerous pores of varying sizes on the cell membrane and cell structure collapse (116). High concentrations of OVA were shown to increase the surface expression of MHC II molecules induce pyroptosis in BMDCs and promote BMDC antigen presentation, inducing an imbalance of Th1/Th2/Th17 cytokines, which directly aggravates allergic inflammation (116).

In summary, NLRP3 inflammasomes, activated by diverse agonists, can induce pyroptosis and the maturation and release of inflammatory mediators IL-1β and IL-18. They also regulate the Th1/Th2 balance and promote the expression of inflammatory factors such as IL-6, IL-17, and IL-33, thereby contributing to allergic disease pathogenesis. Targeting the NLRP3 inflammasome activation pathway or the cleavage process of inflammatory mediators presents a potential therapeutic strategy for allergic diseases.

### 4.4 Other pathways for the role of NLRP3 in allergic diseases

Some research indicates that several alternative pathways can activate NLRP3 inflammasome-mediated pyroptosis, thereby contributing to the development of allergic diseases. Yang et al. discovered that protopine, an isoquinoline alkaloid, can mitigate ovalbumin-induced asthma. The protective mechanism is linked to the suppression of the TLR4/MyD88/NF-κB pathway and the inhibition of NLRP3 inflammasome-mediated pyroptosis (117). Xu et al. demonstrated in the mouse model of asthma induced by house dust mites that knocking out ULK1 gene can reduce the infiltration of inflammatory cells in lung tissue, restore the imbalance of Th1/Th2 ratio, and inhibit the formation of inflammasome, and the researcher suggested that inhibiting the activation of the ULK1/Atg9a/Rab9 signaling pathways can inhibit Golgi apparatus fragmentation and mitochondrial oxidative stress in asthma while reducing the production of NLRP3-mediated pulmonary epithelial inflammation (118). In addition, miRNA regulation of NLRP3 also plays a role in the progression of allergic diseases. Recent studies have isolated and purified exosomes from rat bone marrow mesenchymal stem cells (BMMSCs), which were obtained from ATCC (Manassas, USA)) and found that miR-223–3p, which is highly expressed in BMMSCs-derived exosomes, can improve OVA-induced airway remodeling and inflammation in asthma rats by modulating NLRP3-induced ASC/Caspase-1/GSDMD signaling (119). Similarly, miR-20b targets the TXNIP, suppresses TXNIP expression, and reduces TXNIP binding to NLRP3, thereby limiting pyroptosis and airway inflammation in a murine model of asthma (120). Xiao et al. demonstrated that miR-133b mitigates allergic inflammatory responses and symptoms by targeting NLRP3 in AR mouse models, significantly reducing the expression of NLRP3, Caspase-1, ASC, IL-18, and IL-1β (121). These findings suggest that gene knockout technology to interfere with NLRP3 expression may significantly alleviate the inflammatory symptoms of allergic diseases. Moreover, studies have shown that particulate matter, such as silica nanoparticles, can exacerbate airway inflammation in mouse models of asthma by activating NLRP3 inflammasomes (122). Additionally, PM2.5 exposure has been found to worsen AR symptoms, increase serum IgE secretion, damage the ultrastructure of the nasal mucosa, and significantly upregulate the protein expressions of NLRP3, caspase-1, GSDMD, and IL-1β (13). In summary, the NLRP3 inflammasome can be activated through various mechanisms, pathways, and substances, all of which contribute to the occurrence and development of allergic diseases. Understanding these diverse NLRP3 activation pathways is crucial for developing novel therapeutic strategies for allergic diseases.

## 5 Potential therapeutic approaches for allergic diseases targeting the NLRP3 inflammasome

Current clinical treatments for NLRP3-related diseases, often focusing on IL-1β inhibition, have limitations. While effective in some cases, they may not address all the biological effects triggered by NLRP3 inflammasome activation. Other inflammatory factors released during this process can still contribute to disease progression, leading to suboptimal treatment outcomes and reduced quality of life for patients. This highlights the need for more comprehensive therapeutic approaches. Fueled by the rising global burden of allergic diseases, researchers are increasingly interested in the role of the NLRP3 inflammasome in their development. This interest stems from the potential of the NLRP3 inflammasome as a therapeutic target for allergic diseases, as evidenced by ongoing research on NLRP3 inhibitors (**Table 1**).

**Table 1:** Potential mechanisms of several NLRP3 inflammasome inhibitors in allergic diseases.

	NLRP3inflammasome inhibitor	Potential mechanisms	References
Small molecule inhibitors	MCC950	1.Inhibits the activity of NLRP3. 2.Interfere with chloride efflux.	(123–125)
	OLT1177	1.Directly binds to NLRP3 and inhibits ATPase activity. 2.Prevented NLRP3-ASC, NLRP3-caspase-1 interaction.	(126, 127)
	CY-09	1.Inhibits NLRP3 ATPase activity.	(128)
	Tranilast	1.Inhibition NLRP3 oligomerization. 2.Enhances NLRP3 ubiquitination.	(129)
	Oridonin	1.Blocks the interaction between NLRP3 and NEK7.	(130)
	RRx-001	1.Blocks the interaction between NLRP3 and NEK7.	(131)
Natural products and derivatives	XQLD	1.Inhibits NLRP3 inflammasome-mediated pyroptosis.	(132)
	APS	1.Inhibits the activation of NLRP3. 2.Blocking the phosphorylation of NF-κB. 3.Decreasing NOD2 expression.	(133)
	MFXD	1.Inhibitis the NLRP3/Caspase-1/GSDMD-N signaling pathway.	(134)
	Schisandrin B	1.Inhibits the activation of NLRP3.	(135)
	Houttuynia cordata	1.Decreases the expression of NLRP3, ASC, caspase-1, GSDMD, IL-1β, and IL-18.	(136)
	Angelica Yinzi	1.Inhibiting the activation of the NLRP3. 2.Inhibiting the MAPKs/NF-κB signaling.	(137)

XQLD, Xiaoqinglong decoction.

APS, Astragalus polysaccharide.

MFXD, Mahuang Fuzi Xixin decoction.

### 5.1 Small molecule inhibitors associated with NLRP3 inflammasomes

#### 5.1.1 MCC950

MCC950 is a diarylsulfonylurea-based compound that highly specific inhibits NLRP3 inflammasome activation without affecting AIM2, NLRC4, or NLRP1 inflammasomes. It operates by inhibiting the activities of NLRP3, caspase-1, and ASC, thereby reducing the secretion of IL-1β and IL-18 in various allergic diseases (123, 124). Research by Coll et al. indicates that MCC950 specifically inhibits both canonical and noncanonical NLRP3 inflammasome activation and IL-1β secretion by preventing NLRP3-induced ASC oligomerization in human and mouse macrophages (123). Importantly, MCC950 treatment does not inhibit the priming step of TLR signaling or NLRP3 activation, its inhibition is independent of K^+^ efflux, Ca^2+^ flux, or NLRP3-asc interactions, and MCC950 does not directly inhibit NEK7-NLRP3 or NLRP3-NLRP3 interactions (123). Based on this, the study showed that MCC950 does not appear to affect the initiation step of NLRP3 inflammasome priming, but rather the assembly step of NLRP3 inflammasome (125). Additionally, Zhang et al. observed a significant downregulation of sneezing, nose rubbing, inflammatory cytokines, inflammatory cells, and the expression of NLRP3, caspase-1, ASC, IL-1β, and IL-18 in MCC950-treated mice compared to normal allergic rhinitis mice (124). Wu et al. have identified potential targets of MCC950 during NLRP3 priming and assembly, suggesting that it may interfere with chloride efflux, CLICs, or other targets acting upstream of chloride efflux (125). MCC950 emerges as a promising candidate for the treatment of inflammatory diseases, with its specific inhibition offering a potential therapeutic strategy for allergic diseases involving typical and/or atypical NLRP3 inflammasomes.

#### 5.1.2 OLT1177

OLT1177 is an orally active β-sulfonyl cyanide molecule that has undergone pharmacokinetic and safety analysis in phase 1 trials with healthy volunteers (138, 139). It selectively inhibits the activation of both canonical and noncanonical NLRP3 inflammasomes without impacting AIM2 and NLRC4 inflammasomes. OLT1177’s anti-inflammatory effect is independent of signal 1 (NLRP3 and pro-IL-1β expression) or K^+^ leakage, instead, it directly binds to NLRP3 to inhibit ATPase activity (126). Lunding et al. demonstrated that OLT1177 could inhibit NLRP3 and caspase-1 activity, reduce the release of pro-inflammatory IL-1β, and ameliorate pathophysiological indicators in three experimental mouse models of allergic asthma (127). Marchetti et al. showed that *in vitro*, OLT1177 prevented NLRP3-ASC, as well as NLRP3-caspase-1 interaction at nanomolar concentrations, thereby inhibiting NLRP3 inflammasome oligomerization and reducing the release of IL-1β and IL-18 (126). In LPS-stimulated human blood-derived macrophages, OLT1177 effectively lowered IL-1β and IL-18 levels by 60% and 70%, respectively, with a favorable safety profile for the treatment of acute and chronic inflammation (126). OLT1177’s specific inhibition of NLRP3 inflammasomes offers a promising therapeutic approach to prevent the release of pro-inflammatory factors IL-1β and IL-18, potentially benefiting the treatment of allergic diseases.

#### 5.1.3 CY-09

CY-09 is an analogue of CFTR (inh)-172 (C172), an inhibitor of the cystic fibrosis transmembrane conductance regulator (CFTR) channels (140). Studies have shown that CY-09 specifically blocks NLRP3 inflammasome activation (128). Jiang et al. found that CY-09 directly binds to the ATP-binding motif of the NLRP3 NACHT domain, inhibiting NLRP3 ATP enzyme activity and, consequently, the assembly and activation of NLRP3 inflammasomes. CY-09 directly targets NLRP3, inhibiting its inflammasome activation *in vivo*, and has shown significant therapeutic effects in mouse models of NLRP3-driven diseases, such as type 2 diabetes and CAPS (128). Zhou et al. demonstrated that CY-09 could improve olfactory damage in AR mice by inhibiting NLRP3-mediated pyroptosis and reducing nasal mucosal eosinophil infiltration and goblet cell proliferation (141). CY-09 has a favorable pharmacokinetic profile, with ideal oral bioavailability, safety, and stability (128). It represents a selective and direct small molecule inhibitor of NLRP3, offering potential as a treatment for NLRP3-mediated allergic diseases.

#### 5.1.4 Tranilast

Tranilast, a tryptophan metabolite, is a widely used anti-allergic drug that inhibits NLRP3 inflammasome activation without affecting AIM2 or NLRC4 inflammasomes (129). Notably, tranilast does not interfere with the upstream signaling pathways of NLRP3 inflammasomes, including NLRP3 and pro-IL-1β expression, K^+^ efflux, mitochondrial damage, ROS production, and chloride efflux, nor does it prevent NEK7, a newly discovered inflammasome component of NLRP3, from interacting with NLRP3, but tranilast can directly binds to the NACHT domain of NLRP3 and inhibits the assembly of NLRP3 inflammasomes by blocking NLRP3 oligomerization (129). Chen et al. found that tranilast effectively enhances NLRP3 ubiquitination, attenuated NLRP3 inflammasome assembly and activation, and improved vascular inflammation and atherosclerosis in mice deficient in low-density lipoprotein receptor and apolipoprotein E (142). Tranilast is also recognized for stabilizing mast cell and basophil membranes, preventing degranulation, and inhibiting the release of allergic mediators, offering preventive and therapeutic benefits for bronchial asthma and allergic rhinitis (143).

#### 5.1.5 Oridonin

Oridonin, an ent-kaurane diterpenoid and the primary active component of Rabdosia rubescens, has been shown to be a specific covalent inhibitor of the NLRP3 inflammasome (139). It forms a covalent bond with cysteine 279 in the NACHT domain of NLRP3, blocking the interaction between NLRP3 and NEK7, and inhibiting the assembly and activation of the NLRP3 inflammasome (130). Oridonin selectively inhibits NLRP3 inflammasomes without affecting AIM2 or NLRC4 inflammasomes, LPS-induced NLRP3, pro-IL-1β expression, or TNF-α production (130). Wang et al. found that Oridonin treatment significantly reduced the expression of IL-4-induced cleaved caspase-1, ASC, and NLRP3 proteins in an *in vitro* pediatric asthma model, suggesting its potential as a novel therapeutic agent for NLRP3-mediated allergic asthma (144). In addition, it has been shown to participate in the prognosis and treatment of NLRP3-related diseases by directly targeting NLRP3, as demonstrated in mouse models of traumatic brain injury and myocardial infarction, where it has been found to reduce inflammation, prevent neuronal apoptosis, maintain the blood-brain barrier, alleviate neurological deficits, inhibit myocardial fibrosis, reduce infarction size, and improve cardiac function (145, 146). As a clinical NLRP3 inflammasome inhibitor, Oridonin holds promise for future applications in the treatment of allergic diseases.

#### 5.1.6 RRx-001

RRx-001 is a pleiotropic anticancer agent currently in phase III clinical trials (147). Studies have shown that RRx-001 significantly inhibits HDM-induced eosinophil, neutrophil, and lymphocyte infiltration of the airways and improves allergic asthma symptoms by inhibiting NLRP3 activation and eosinophilic airway inflammation (104). Chen et al. found that RRx-001 is a highly selective and potent NLRP3 inhibitor that inhibits the activation of canonical, non- canonical, and alternative NLRP3 inflammasomes without affecting AIM2, NLRC4, or Pyrin inflammasomes, showing significant efficacy in NLRP3-driven inflammatory diseases (131). RRx-001 was found to covalently bind to cysteine 409 of NLRP3 through its bromoacetyl group, blocking the NLRP3-NEK7 interaction and subsequently inhibiting the assembly and activation of the NLRP3 inflammasome (131). With further research, RRx-001 may emerge as a specific therapeutic small molecule for the treatment of NLRP3-mediated allergic diseases.

### 5.2 Natural products and derivatives act as NLRP3 inflammasome inhibitors

The exploration of natural medicinal plant ingredients has developed as an increasing number of medicinal plants demonstrate effective anti-inflammatory properties against inflammatory diseases. Recent research has identified natural small molecules with the potential to inhibit NLRP3 inflammasome activation, which can act as potential treatments for allergic diseases. Bai et al. discovered that water-extracted Lonicera japonica polysaccharide (WLJP) mitigates allergic rhinitis symptoms by modulating the NLRP3-IL-17 signaling axis. Experimental evidence suggests that WLJP can reduce serum inflammatory factors, eosinophil counts, and goblet cell hyperplasia. It also decreases NLRP3 inflammasomes, Th17 cell differentiation in the spleen, and the expression of IL-17, p-p65, and intestinal NLRP3 in the nasal mucosa, thereby maintaining the stability of the barrier function (148). Similarly, in the establishment of a BALB/C model of AR mice using ovalbumin (OVA) and aluminum hydroxide sensitization, Xiaoqinglong decoction (XQLD) has been shown to significantly alleviate nasal allergy symptoms and reduce the proliferation and infiltration of inflammatory cells in the nasal mucosa. It downregulates the expression of Th2 inflammatory factors (IL-4, IL-5, IL-13) in serum and nasal mucosa and markedly decreases the expression of IL-1β and IL-18. Molecular docking studies indicate that seven representative compounds of XQLD exhibit favorable binding properties to NLRP3, potentially inhibiting NLRP3 inflammasome-mediated pyroptosis in nasal mucosa and improving allergic rhinitis symptoms (132). Furthermore, in a rat model of AR, research shows that Astragalus polysaccharide (APS) not only inhibited the NLRP3 inflammasome activation but also inhibited NF-κB activation by decreasing NOD2 expression and blocking the phosphorylation of NF-κB (133). Similarly, In the AR mouse model, Mahuang Fuzi Xixin decoction (MFXD) suppressed nasal epithelial pyroptosis by inhibiting the NLRP3/Caspase-1/GSDMD-N signaling pathway, which alleviates AR (134).

Natural medical plants are also playing a therapeutic role in the pathological processes of NLRP3-mediated allergic asthma. Chen et al. demonstrated that *Schisandrin B* inhibits NLRP3 inflammasome activation and pyroptosis through the miR-135a-5p/TRPC1/STAT3/NF-κB axis, thereby reducing airway inflammation and remodeling in asthma (135). *Houttuynia cordata* has been found to possess pharmacological properties that reduce airway inflammation and reactivity in asthma. Treatment with Houttuyndin sodium decreases the expression of NLRP3, ASC, caspase-1, GSDMD, IL-1β, and IL-18 in mouse lung tissue, alleviating NLRP3-related pyroptosis and Th1/Th2 immune imbalances, and thus reducing inflammatory responses (136). Additionally, the application of NLRP3 natural product inhibitors in AD has been further investigated. Liu et al. found that *Angelica Yinzi* (AYZ) could inhibit the activation of the NLRP3 inflammasome and MAPKs/NF-κB signaling, effectively downregulating the expression of NLRP3, ASC, Caspase-1, and IL-1β in the dorsal skin of mice after treatment, and effectively suppressing AD-induced skin inflammation (137). AYZ is thus considered a potential novel therapeutic agent for the clinical treatment of AD. As our understanding of the pharmacological effects of natural plants deepens, more studies are targeting the mechanisms of NLRP3 in allergic diseases. The aim is to discover potential and effective natural pharmaceutical ingredients for disease treatment.

## 6 Conclusion

This review has illuminated the critical role of NLRP3 inflammasome-mediated pyroptosis in allergic diseases, delving into the intricate mechanisms by which NLRP3 activation fuels inflammation and exacerbates allergic symptoms. The recent breakthroughs in understanding the NLRP3 inflammasome have opened a new frontier for therapeutic development. Promising avenues include developing NLRP3 inhibitors and modulators of downstream pyroptosis pathways, as well as neutralizing key inflammatory mediators. However, translating these discoveries into clinical practice requires further research: (1) Clinical trials to ensure safety and efficacy in humans, (2) refined targeting through a deeper understanding of NLRP3 inflammasome function within the nasal cavity, and (3) personalized medicine to identify responders to NLRP3-based therapies. By overcoming these challenges and further elucidating the NLRP3 inflammasome’s role in allergies, we can unlock a new era of therapeutic strategies that not only manage symptoms but also prevent the underlying inflammatory processes driving these conditions, offering significant relief to allergy sufferers.

## Author contributions

HL: Writing – original draft, Formal analysis, Data curation. YZ: Writing – original draft, Formal analysis, Data curation. TH: Writing – review & editing. DY: Writing – review & editing. XW: Writing – review & editing. DL: Writing – review & editing. SQ: Writing – review & editing, Supervision, Funding acquisition. BC: Writing – review & editing, Supervision, Project administration, Methodology, Funding acquisition. XZ: Writing – review & editing, Supervision, Project administration, Funding acquisition.
